# Early Surgical Intervention for Neovascular Glaucoma in a Patient with Diabetes

**DOI:** 10.7759/cureus.15420

**Published:** 2021-06-03

**Authors:** Khatoon A Husain, Haneen Alaali, Husain Alderazi

**Affiliations:** 1 Ophthalmology Department, Salmaniya Medical Complex, Manama, BHR

**Keywords:** ophthalmology, neovascular glaucoma, glaucoma surgery, anti-vegf treatment, diabetic eye disease, pan-retinal photocoagulation, diabetes mellitus

## Abstract

Neovascular glaucoma (NVG) is a cause of blindness in patients with proliferative diabetic retinopathy (PDR) and remains a clinical challenge. It results from vascular endothelial growth factor (VEGF) expression, which occurs in cases of severe retinal ischemia. Early detection and immediate comprehensive management, including early surgical intervention, are essential to maintain good intraocular pressure (IOP) control and achieve the best visual outcome, as presented in this case. A 42-year-old male patient with bilateral NVG secondary to PDR received complex management with early Ahmed valve implantation surgery, followed by pan-retinal photocoagulation (PRP) and intravitreal bevacizumab injection. After 22 months of treatment and follow-up, we could achieve the best visual outcome for the patient. Early surgical intervention for uncontrolled NVG with a glaucoma filtration device combined with intravitreal anti-VEGF injections and adequate PRP showed beneficial effects in the IOP control with rapid resolution of iris neovascularization, thus achieving the best visual function.

## Introduction

Neovascular glaucoma (NVG) is a secondary glaucoma that causes vision loss and is characterized by the development of neovascularization of the iris (NVI), elevated intraocular pressure (IOP), and, in many instances, poor visual prognosis [1]. NVG results from severe ocular ischemia and is commonly associated with posterior segment conditions such as proliferative diabetic retinopathy (PDR), central retinal vein occlusion (CRVO), central retinal artery occlusion, and ocular ischemic syndrome [2]. The estimated incidence of NVG in patients with diabetes is approximately 6.6/10,000 individuals, which accounts for 5% of blindness cases in these patients [1]. The term NVG was first used by Pagenstecher in 1871 and was used to describe hemorrhagic, thrombotic, congestive, rubeotic, and diabetic hemorrhagic glaucomas [3,4].

The association between NVI and diabetes mellitus was first reported by Salus in 1928, and PDR was found to be a major cause of NVG, representing approximately 30% of cases [5]. NVG remains a potentially sight-threatening condition and is usually refractory to medical treatment. Moreover, the visual prognosis can be guarded as these patients have uncontrolled systemic conditions that increase the progression of ischemia [5]. Thus, early diagnosis and aggressive medical management with early surgical intervention are essential to prevent vision loss and improve prognosis.

We present a case of bilateral NVG secondary to PDR that was challenging to treat. In the presented case, early surgical intervention was essential to maintain the best visual function.

## Case presentation

Medical and ophthalmic patient history

A 42-year-old male patient who had a known case of poorly controlled type 1 diabetes and hypertension presented to our ophthalmology emergency department on July 1, 2019, with a four-day history of painful, acute vision loss in his right eye (OD). He had no history of trauma or previous eye surgeries and had never undergone an ophthalmological examination.

Examination revealed a vision of 6/36 in the right eye and 6/9 in the left eye (OS). The right eye had a conjunctival injection and corneal edema with 360° of rubeosis iridis (NVI). The left eye showed a clear cornea with subtle vessels at the pupillary margin. His IOP was 52 mmHg in the right eye and 12 mmHg in the left eye. Dilated fundus examination (DFE) of the right eye was limited due to corneal edema, but it was highly suggestive of PDR. DFE of the left eye fundus showed a clear vitreous with PDR.

Gonioscopy revealed Shaffer grade 1 over 360° with neovascularization of the angle OD and Shaffer grade 3 over 360° and no neovascularization of the angle in the left eye.

Clinical course

On initial presentation, the patient was diagnosed with NVG OD with a high-risk PDR in both eyes (OU). The patient was admitted to the hospital. For his elevated IOP, he was treated with intravenous mannitol (20%) and oral acetazolamide, along with anti-glaucoma drops such as brimonidine, timolol, and dorzolamide. Topical steroids and cycloplegic drops (atropine) were also administered. He received an intravitreal injection of bevacizumab (1.25 mg). His IOP was still above 40 mmHg and pan-retinal photocoagulation (PRP) could not be performed due to corneal edema; therefore, he underwent Ahmed valve implantation OD within one week of presentation.

During his hospital stay, his clinical course was followed up by an endocrinologist to ensure tight glycemic control, with an HbA1c target of 6%.

The postoperative course was uneventful and IOP was kept under control. Multiple sessions of PRP were performed in both eyes, along with additional bevacizumab injections. Treatment with aqueous suppressant eye drops was started three weeks after surgery despite his normal IOP to reduce the frequency of the hypertensive phase. The patient adhered to the therapy and maintained a good visual acuity of 6/9 OD and 6/6p OS for six months despite his rapidly progressing disease. Unfortunately, he was lost to follow-up as he traveled overseas.

The patient again visited our ophthalmology emergency department in April 2020 with a marked decrease in vision to hand motion OS for one week. On examination, he was noted to have IOP and NVI of 61 mmHg and 360°, respectively. The IOP of the right eye, which was operated, was 16 mmHg, and the vision was 6/9 despite having active NVI. He was hospitalized and treated with anti-glaucoma eye drops along with intravenous mannitol 20% and oral acetazolamide. After three treatment cycles, his IOP remained very high, compromising the retinal visualization and further treatment of his active PDR. Therefore, he underwent urgent Ahmed valve implantation OS with ranibizumab intravitreal injection on April 3, 2020. He needed an anterior chamber washout to clear his NVI-related hyphema on April 16, 2020, following which his IOP was well controlled with two anti-glaucoma drops.

**Figure 1:**
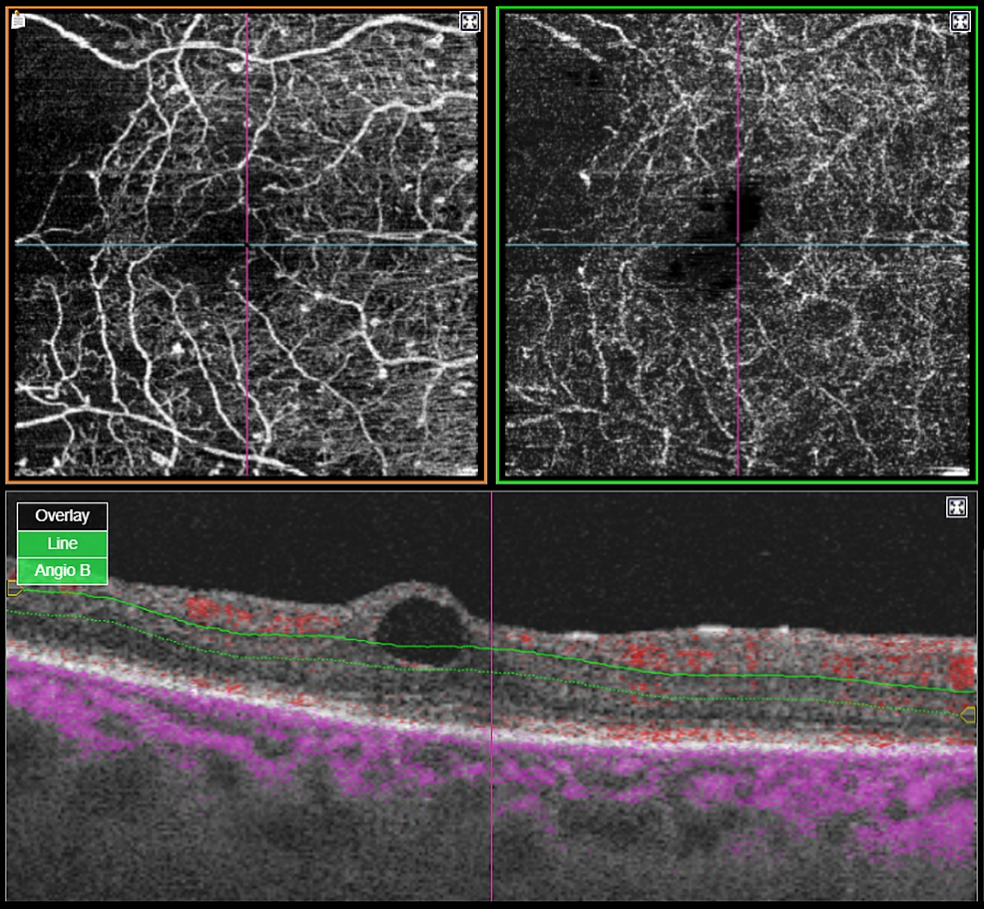
Optical coherence tomography angiography (4.5 × 4.5 mm) of the right eye shows a macular cyst.

**Figure 2:**
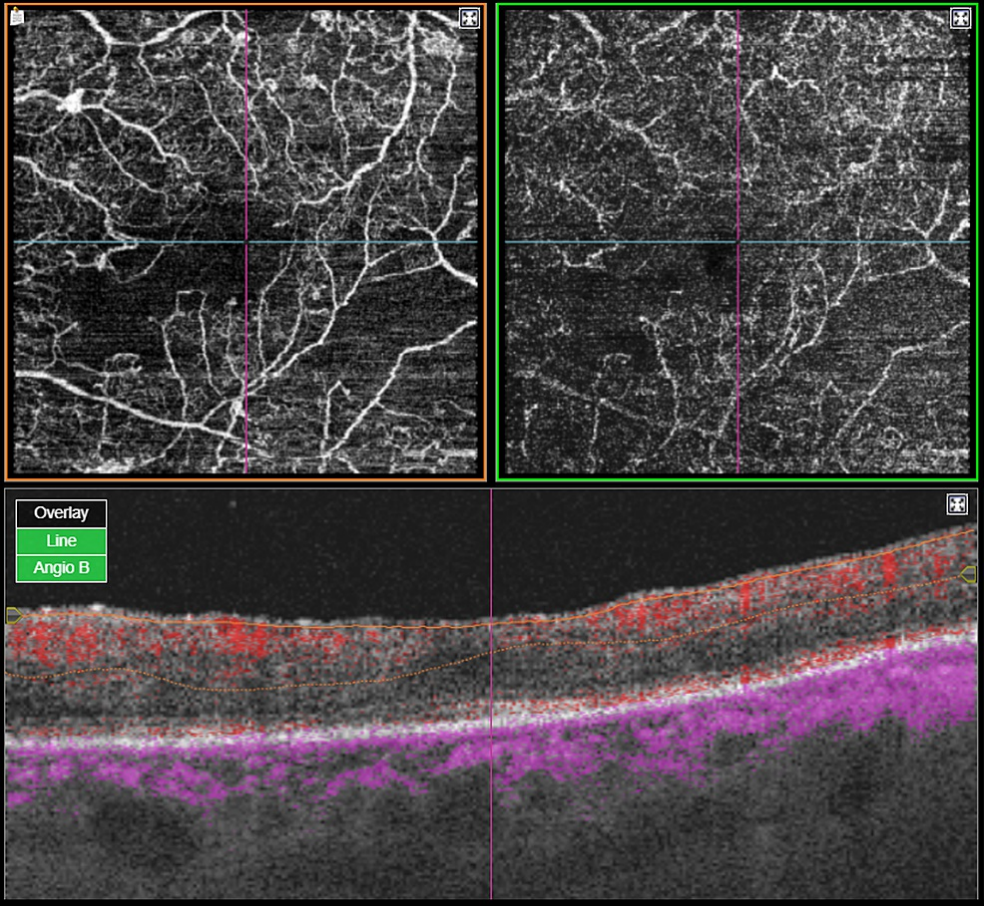
Optical coherence tomography angiography (4.5 × 4.5 mm) of the left eye shows an enlarged foveal avascular zone.

At that time, the patient underwent several PRP sessions, with 3,200 burns to the right eye and 3,500 to his left eye, in addition to multiple injections of anti-vascular endothelial growth factor (anti-VEGF), allowing for rubeosis regression. Optical coherence tomography angiography revealed an enlarged foveal avascular zone (OS) which probably might explain the reduction of vision in his left eye (Figures 1, 2).

**Figure 3:**
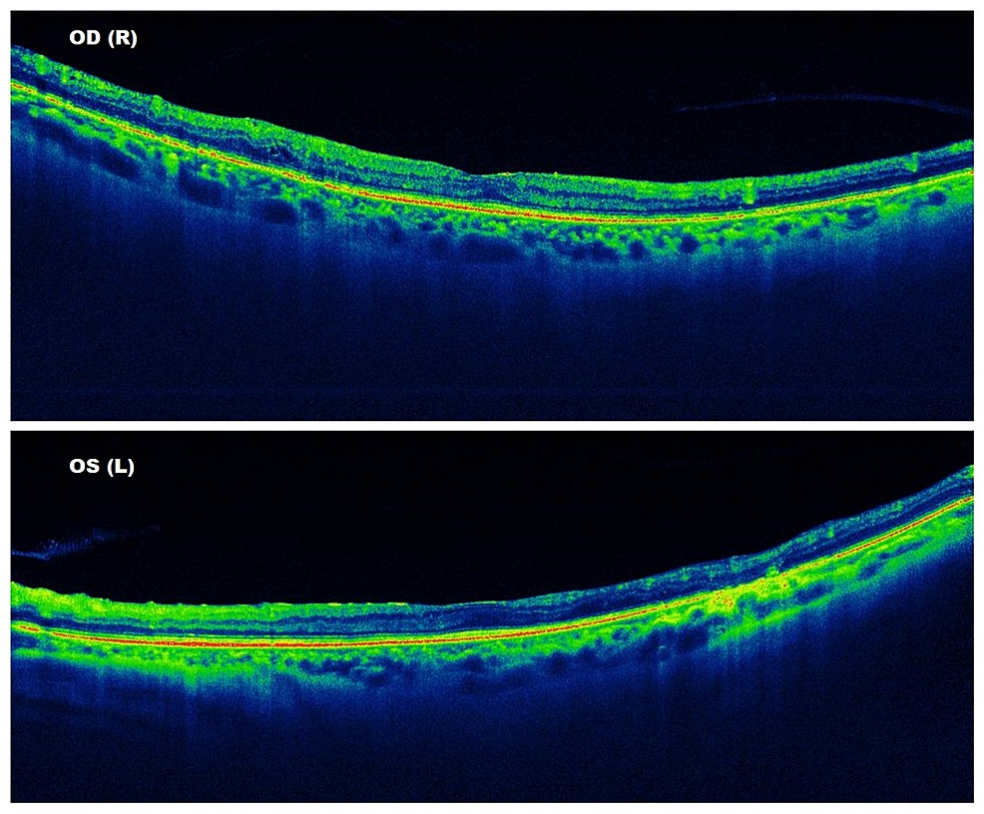
Macular 12-mm radial B scan optical coherence tomography shows a dry macula bilaterally.

**Figure 4:**
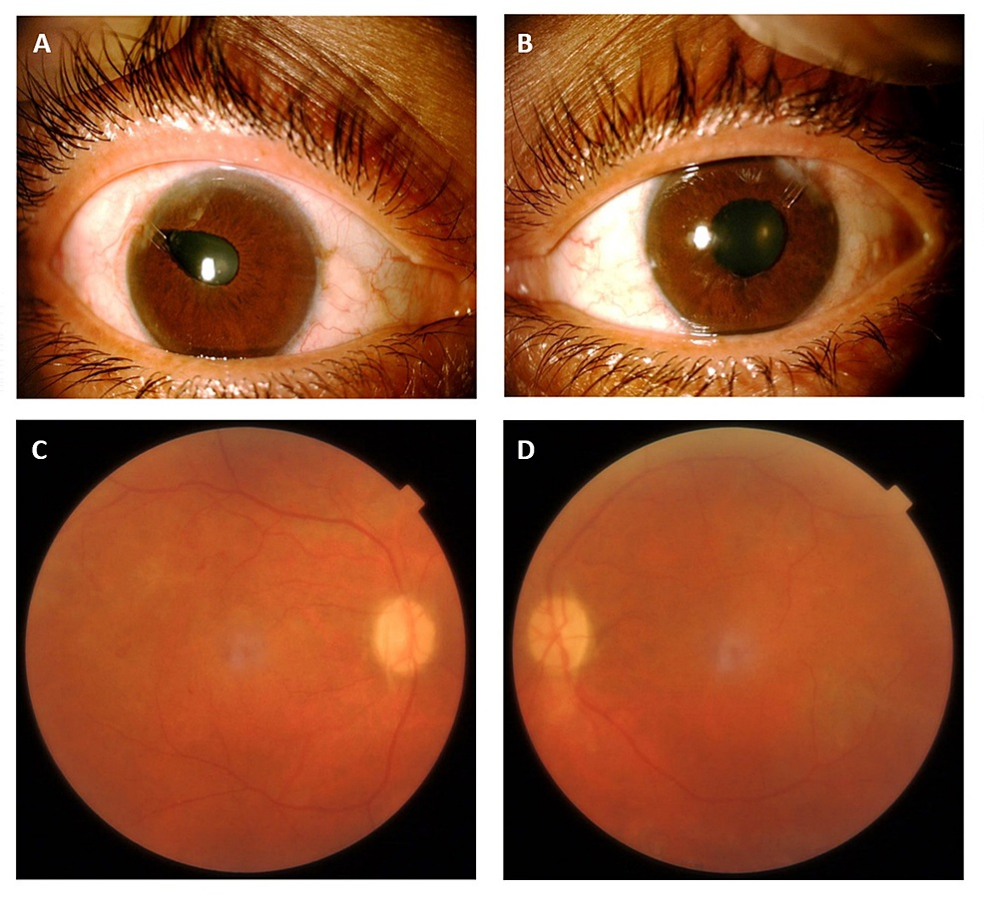
(A) Right eye slit lamp image showing regressed NVI and AVI. (B) Left eye slit lamp image showing regressed NVI and AVI. (C) Right eye color fundus image. (D) Left eye color fundus image. AVI: Ahmed valve implant; NVI: neovascularization of the iris

During his last outpatient clinic visit, his visual acuity was 6/6 OD and 6/60 OS. His IOP was 14 mmHg OD and 16 mmHg OS. He had stable diabetic retinopathy with dry macula and full PRP OU (Figures 3, 4).

## Discussion

Pathophysiology of neovascular glaucoma

NVG develops secondary to new vessel formation over the iris and the iridocorneal angle, which leads to the obstruction of aqueous humor outflow and increased IOP. It can present with an open or closed angle when the fibrovascular membrane contracts, causing synechial closure [6].

Although diabetes is the causative factor in 30% of cases, yearly screening has helped reduce this proportion, which now occurs in 10-15% of cases. Currently, the major cause of NVG is CRVO, which accounts for 35-50% of cases. Other causes include arterial retinal vascular disease, intraocular tumors, long-standing retinal detachment, and chronic intraocular inflammation [2].

In most cases, the pathogenesis is related to posterior segment ischemia. In ischemic retinas, such as those seen in patients with diabetes, there is an imbalance between pro-angiogenic factors (such as VEGF) and other anti-angiogenic factors (such as pigment-epithelium-derived factor), leading to neovascularization [7].

In patients with poorly controlled diabetes and extensive untreated retinal neovascularization, progression from NVI to NVG is frequent. The time interval between the onset of NVI and NVG in untreated cases varies from one month to over three years [8].

Diagnosis and clinical investigations

The diagnosis of NVG can be made clinically, although it is difficult to recognize in the early stages. It is essential to examine the iris before pupil dilatation to detect early NVI. In addition, careful gonioscopy is mandatory for neovascularization of the angle and synechia.

Management options

NVG needs comprehensive management; in addition to the administration of retinal and glaucoma care, the systemic condition should be addressed to protect the other eye in unilateral cases and to decrease the progression of ischemia.

Patients with diabetes require tight glycemic control and a proper systemic workup to prevent further complications. In addition, ocular treatment should be initiated immediately. This includes medical therapy, PRP, anti-VEGF injections, and surgical intervention.

Medical therapy

The choice of IOP-lowering agents depends on the stage of NVG and visual recovery potential. Patients with good visual potential and more advanced cupping require a lower target IOP. Because of the inflammatory nature of this condition, the choice of anti-glaucoma drops is limited. Any of the following aqueous suppressant medications can be used: topical beta-blockers (e.g., timolol 0.5%), carbonic anhydrase inhibitor (e.g., dorzalamide 2%), and topical alpha 2 agonists (e.g., brimonodine 1% to 0.2%), which may be effective in lowering IOP when used in combination. Prostaglandin analogs may help lower IOP; however, they may increase inflammation and should be avoided in the acute phase [2].

Systemic therapy includes hyperosmolar agents (e.g., mannitol 20%) and oral carbonic anhydrase inhibitors. These agents can be used when the IOP cannot be controlled with topical drops. However, renal function should be monitored regularly, especially in patients with concomitant diabetic nephropathy [2].

In addition, adjuvant topical steroids and cycloplegic agents such as atropine may control inflammation, relieve discomfort, and lower the pressure by increasing uveoscleral outflow and reducing the incidence of hyphema. Despite these measures, failure of medical therapy alone is not uncommon, especially in grade 3 NVG with synechial angle closure [6].

Pan-retinal photocoagulation and anti-vascular endothelial growth factor therapy

PRP should be employed in all cases of NVG when the etiology is retinal ischemia as it is the mainstay in controlling neovascularization. Extensive PRP is indicated for severe ischemic retinopathy, which helps in the regression of neovascularization and in lowering IOP [9]. However, adequate PRP might not be possible due to corneal edema secondary to high IOP, as seen in our case. In addition, PRP can lead to an increase in IOP from cilio-choroidal effusions [10], adding more compromise to the optic nerve, especially in cases of uncontrolled IOP. In this situation, the IOP should be controlled first, and if possible, intraocular anti-VEGF injections may be administered as adjuvant therapy.

The administration of anti-VEGF has helped NVG management evolve and is currently supported by several studies suggesting better visual prognosis and IOP control following anti-VEGF injections [7]. However, due to the temporary nature of anti-VEGF injections, it should be combined with adequate PRP. Gheith et al. suggested that intravitreal bevacizumab (IVB) may be a valuable addition in the treatment of NVG by hastening the resolution of anterior segment neovascularization, thereby improving the results of glaucoma surgeries, and it appears to provide long-term control when used in combination with PRP [11].

In addition, intravitreal anti-VEGF injection, administered before surgical intervention, helps regress neovascularization and improve the success rate of aqueous shunts. This finding is supported by that reported in another study which concluded that preoperative IVB before Ahmed glaucoma valve surgery decreased the risk of postoperative hyphema [12]. In our case, the presurgical injection of IVB in the right eye reduced the risk of intraoperative and postoperative bleeding.

Surgery

As mentioned earlier, surgical interventions to control IOP are often necessary as the use of topical therapy might not be adequate, especially in synechial angle-closure NVG as in our case. Surgical options include trabeculectomy with mitomycin C, a glaucoma drainage device, and ciliary body destructive surgery. Among these modalities, glaucoma drainage implants have become the primary option in NVG management for many surgeons because of the low success rates reported in conventional trabeculectomy surgery and the severe complications associated with ciliary body destructive surgery [13,14].

The Ahmed valve has become more popular among drainage devices as it has a one-way pressure-sensitive valve, and it reduces the rate of hypotony after surgery. The surgical success rate in patients with NVG ranges from 22% to 97% [15].

Close follow-up after surgery is crucial to enhance the success rate as the IOP may increase during the hypertensive phase, which tends to occur one to three months after surgery [16].

Some clinical studies support the initiation of topical aqueous suppressant eye drops before the onset of the hypertensive phase as it improves IOP reduction, the frequency of the hypertensive phase, and the success rate [17]. In our case, this was applied, and the patient maintained good IOP control despite his progressive disease.

Other factors that might increase the failure rate after surgery include inadequate retinal photocoagulation therapy [18] and progression of primary vascular diseases. Mermoud et al. suggested that surgical effects in patients with diabetes were superior to those in patients with CRVO [19].

Therefore, retina specialists should provide adequate retinal photocoagulation after the implantation of Ahmed valve drainage for patients with NVG. This consequently promotes retinal angiogenesis regression and enhances surgical success.

## Conclusions

NVG secondary to PDR remains a challenge to treat and has a guarded prognosis. However, a multidisciplinary approach with tight glycemic control and early surgical intervention with glaucoma drainage devices, along with adequate PRP and intraocular anti-VEGF injections, showed good long-term outcomes in terms of resolution of NVI and IOP control, which, in turn, resulted in the preservation of visual function.

We presented this case to highlight the importance of early surgical intervention to adequately control the IOP in NVG secondary to PDR and, consequently, help improve the prognosis of the condition and maintain good visual function. Moreover, IOP control will allow prompt laser treatment to the retina to control retinal ischemia, which is the main culprit in cases of NVG.
